# Prediction of the treatment response in ovarian cancer: a ctDNA approach

**DOI:** 10.1186/s13048-020-00729-1

**Published:** 2020-10-19

**Authors:** Mina Sharbatoghli, Somayeh Vafaei, Hamidreza Aboulkheyr Es, Mohsen Asadi-Lari, Mehdi Totonchi, Zahra Madjd

**Affiliations:** 1 Oncopathology Research Center, Iran University of Medical Sciences, Tehran, Iran; 2 Department of Stem Cells and Developmental Biology, Cell Science Research Center, Royan Institute for Stem Cell Biology and Technology, ACECR, Tehran, Iran; 3 Department of Molecular Medicine, Faculty of Advanced Technologies in Medicine, Iran University of Medical Sciences, Tehran, Iran; 4 School of Biomedical Engineering, University of Technology Sydney, Sydney, 2007 Australia; 5 Department of Epidemiology, School of Public Health, Iran University of Medical Sciences, Tehran, Iran; 6 Department of Genetics, Reproductive Biomedicine Research Center, Royan Institute for Reproductive Biomedicine, ACECR, Tehran, Iran

**Keywords:** Ovarian cancer, Circulating tumor DNA, Prognosis

## Abstract

**Abstract:**

Ovarian cancer is the eighth most commonly occurring cancer in women. Clinically, the limitation of conventional screening and monitoring approaches inhibits high throughput analysis of the tumor molecular markers toward prediction of treatment response. Recently, analysis of liquid biopsies including circulating tumor DNA (ctDNA) open new way toward cancer diagnosis and treatment in a personalized manner in various types of solid tumors. In the case of ovarian carcinoma, growing pre-clinical and clinical studies underscored promising application of ctDNA in diagnosis, prognosis, and prediction of treatment response. In this review, we accumulate and highlight recent molecular findings of ctDNA analysis and its associations with treatment response and patient outcome. Additionally, we discussed the potential application of ctDNA in the personalized treatment of ovarian carcinoma.

**Graphical abstract:**

ctDNA-monitoring usage during the ovarian cancer treatments procedures. 
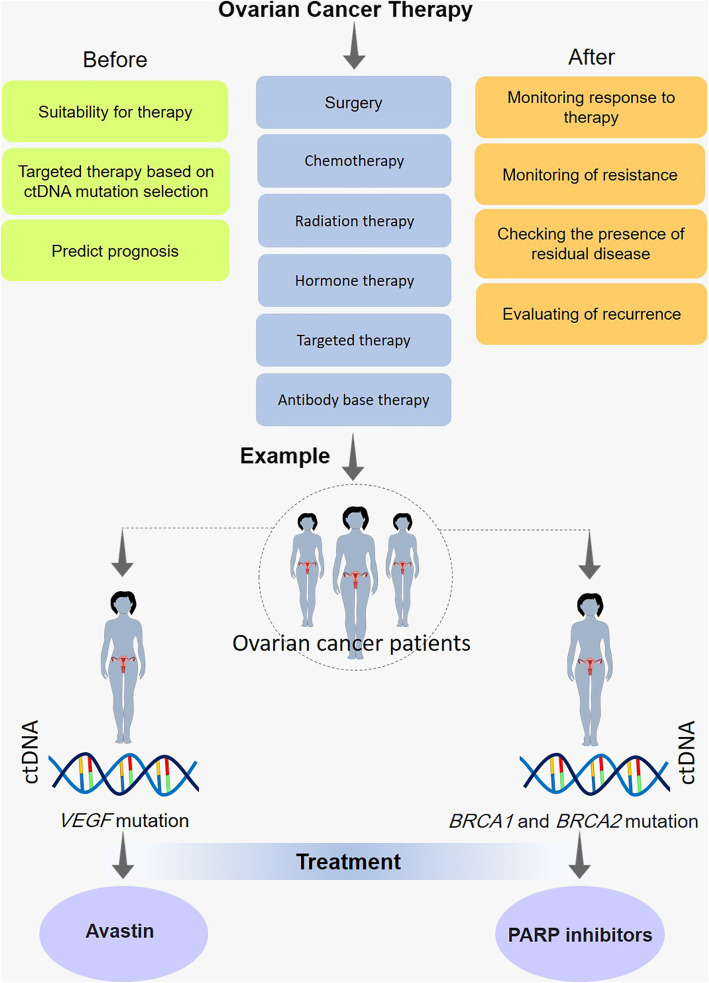

## Introduction

Ovarian cancer was reported with the highest mortality rate **(**almost 50% of new cases are annually reported by the American cancer society) among the gynecologic malignancies [1]. Most of ovarian cancer patients are diagnosed at the advanced stages, at which the tumors have disseminated. Depending on the stages of the disease, the treatment approach may consist of surgery, chemo, radiation, hormone or targeted therapy toward shrinkage and the elimination of the primary tumor and also suppression of the metastatic progression [2]. Clinically, response to standard of the treatment regimens is vary among the patients due to the complexity of disease, particularly cellular and molecular heterogeneities of tumor. Therefore, the prediction of the treatment efficacy at early stages of therapy can enhance the accuracy of the patient’s selection toward the administration of appropriate treatment regimens, and particularly chemotherapy [2]. Although implementation of liquid biopsy approaches improved the patient outcome in various types of cancers including lung and breast in a personalized manner, in the case of ovarian cancer, utilizing an appropriate approach to identify the proper treatment for the specific patient is the matter of debate [3]. Accordingly, to tackle this problem, more studies must be performed to identify the biomarkers responsible for patient response to chemotherapies such as CA125 (Cancer Antigen 125) level [4] or human epididymis protein 4 (HE4) [5]. However, the accuracy and effectiveness of these biomarkers on the prediction of the chemotherapy response differ among the patients with various epidemic and clinical features [6]. Genomic profiling of tissue biopsy provides a snapshot of the dynamic behavior of tumor information and uncovers the genomic characterization of the tumor at the time of diagnosis [7]. Also, these challenges are particularly obvious in the patients who are resistant to therapy or in the patient’s follow up [8]. In recent years, identification and characterization of cancer-derived components such as circulating tumor cells (CTC), exosomes, and circulating tumor DNA (ctDNA) known as liquid biopsy [8, 9], opened a new way in the patients’ stratification and personalized treatment [7]. Of these, the detection of ctDNA tumor-specific mutations show a great promise in the patient’s selection and precision medicine, and besides, it can be suggested as a prognostic factor for the prediction of treatment response across several tumor types including lung, breast, colorectal, and melanoma cancers [9]. The previous review articles have reported the technological aspects of both detection and isolation of CTC alongside ctDNA as a diagnostic marker in ovarian cancer [10, 11]. While the present review, aim to consider mainly the clinical application of ctDNA in treatment and management of ovarian cancer patients. Moreover, the current review presents studies that compare genetic and molecular changes in ctDNA with tissues in ovarian cancer patients. The potential application of ctDNA as a prognostic factor for the prediction of the patient outcome in ovarian cancer also will be discussed.

### The current challenge in the treatment of ovarian cancer

Understanding the molecular mechanisms of ovarian cancer, as a heterogeneous disease and underlying treatment resistance, can lead to discovery of some new therapeutic agents [12]. The standard of care treatment approaches for advance ovarian cancer is relaying on a primary cytoreductive surgery. Accordingly, it can be followed using an adjuvant therapy based on various chemotherapy-accompanied with the combination therapies regimens, which can improve response and the patients overall survival (OS) [13]. Unfortunately, chemotherapy resistance at the cancer advanced-stage is an important clinical challenge [14]. To date, several mechanisms of drug resistance have been explored, including inactivation of the p53 pathway [15], genome wide mutations [16], the enhanced expression of anti-apoptotic genes [17], epigenetic changes [18], dysfunctionality of DNA repair pathways [19], diminished drug accumulation [20], and the elevated drug inactivation [21]. In this regard, all these mechanisms lead to genomic instability, which allows cancer cells to adapt and survive against chemotherapy [22]. Beside these mechanisms, a particular role of cancer stem cells (CSCs) clones are defined within tumor microenvironment (TME) [23] and tumor-associated mesenchymal stem cells [24]. Also it has been observed that, CSCs have an appositive association with platinum, carboplatin, and paclitaxel resistance at the advanced-stage of ovarian cancer. In addition, the TME features including immune cell infiltration, angiogenesis, and hypoxia have been implicated in the platinum chemoresistance [25]. Furthermore, the investigation of molecular ovarian cancer tissue signature has paved the new way for biomarker discovery to assist the clinicians in making better treatment decisions. In this regard, a large number of clinical and preclinical studies suggested potential of tissue-based transcriptomics and proteomics biomarker information for the chemo-resistance prediction [26]. Meanwhile, pre-mediated cellular mechanisms such as clinical phenotypes, chaperones, metabolic proteins, transcription regulators, transporters, and cytoskeletal proteins are up-regulated in the patients with chemo-resistance. Although tissue biopsy is a gold standard to assess the pathological feature of disease, a recently liquid biopsy presented various advantages over this conventional approach [27]. The analysis and role of blood-based biomarker for evaluating the patient response to chemotherapy are undeniable [13]. Also, the plasma-derived ctDNA is the most commonly candidate in clinical practice due to its abundance and overcome on the isolation challenges [28]. The use of ctDNA in monitoring the patients with cancer prevents the risks associated with the repeated tissue biopsies [29].

### The current prognostic biomarkers in the prediction of treatment response in ovarian cancer

Lack of an appropriate approach in chemotherapy response prediction in ovarian cancer is likely leading to poor patient’s survival [30]. CA125, which is expressed by epithelial ovarian tumors and other tissues of mullerian origin, was the first ovarian cancer biomarker described by Rober Bast et al. Accordingly, increase in the serum level of CA125 is observed in diverse malignancies, menstruation or pregnancy, and benign gynecological conditions [31]. The measurement of serum CA125, as a vital biomarker in clinical practice, was applied for screening high risk women, and also to predict clinical course and response to chemotherapy. In fact, dynamic changes in serum CA125 levels as a chemo responsiveness predictor can also be used to predict the response to the first-line and the second-line chemotherapy. However, the clinical value of CA125 is uncertain due to its limitations. For instance; it has been evidenced that, CA125 level is not elevated in 50% of the stage I women and in 30% of more advanced ones [32].

HE4 (Human Epididymis Protein 4) is overexpressed in ovarian cancer. Also, the combination of CA-125 and HE4 have the highest sensitivity and specificity in patients sera and also helps in the prediction of malignancy [33]. In addition, it is important to consider its potential false-positive results with CA 125 [34].

Ova1 measures five proteins named as CA-125, transthyretin, apolipoprotein A1, beta-2 microglobulin, and transferrin with the FDA approval [35]. Vascular Endothelial Growth Factor (VEGF) is a glycosylated angiogenesis mediator, which is independently associated with a shorter OS and disease-free survival. Notably, the combination of VEGF with CA-125 and HE4 increased the diagnostic sensitivity up to 84% at the stage I [36].

Kallikreins (KLKs) with 15 family members are responsible in cell growth, angiogenesis, invasion, and metastasis [37]. The decreased incidences of high “false negative” rates were also found in the HE4 and CA-125 positive patients [38].

Osteopontin (OPN) is a secreted extracellular matrix glycoprotein, which is involved in wound healing, the immune response, inflammation, tumorigenesis, bone remodeling, and apoptosis inhibition [39].

Mesothelin is a cell surface glycoprotein that are important in tumor metastasis, cancer cell survival, proliferation, and drug resistance [40]. Also, Mclntosh et al. detected the increased level of serum mesothelin in 60% of ovarian cancer patients with 98% specificity. So, a combination of mesothelin and CA-125 was suggested [41]. Additionally, Obulhasim et al. found that, mesothelin is expressed in 100% of serous cystadenocarcinoma as well as serous borderline ovarian tumor [42].

In addition to these biomarkers, few prognostic biomarkers were introduced as valuable indicators including macrophage colony-stimulating factor (M-CSF), bikunin, EphA2, Transthyretin (TTR), Transferrin receptor 1, B7-H4, Prostasin, and soluble EGF receptor [43–51] (Table 1).

**Table 1:** List of known biomarkers in prediction of ovarian cancer treatment response

Biomarkers	utility	Weakness
CA125	Can be assessed in epithelial, endometrial and clear cell types in patients with clinical stage I- IV [52].	• Cannot be elevated in some ovarian cancer patients. • Can be elevated in healthy premenopausal women during menses, in pregnancy, in nonmalignant gynecologic diseases, such as ovarian cysts, endometriosis, adenomyosis, and uterine leiomyomas, in several nonmalignant nongynecological diseases, such as peritoneal, pleural, and musculoskeletal inflammatory disorders as well as pelvic inflammatory disease, liver, and renal as well as cardiac disease and in most types of advanced adenocarcinomas, including breast, colorectal, pancreas, lung, endometrium, and cervix as false positive. • Is not expressed in pure mucinous tumors [53].
HE4	Can be assessed in epithelial ovarian adenocarcinomas high [54].	• Can be elevated in endometrioid and clear cell histology [55]. • Cannot be detected in epithelial/ nonepithelial ovarian cancer, including sex cord stromal tumors and germ cell tumors [54] • Overexpressed in gastric cancer, pancreatic cancer as well as occasionally in colon and hepatocellular cancer [56, 57].
Ova1	Ova1 score ≥ 5 in premenopausal women and ≤ 5 postmenopausal ones were detected, and was considered with higher risk of malignancy [58].	• Ova1 demonstrated 92.5% sensitivity, but lower specificity of 42.8% [58]
VEGF	VEGF level was independently associated with shorter disease-free survival and overall survival [59].	• Can be compared with traditional biomarkers, such as CA125 and HE4 moderately [60]. • It must be combined with CA-125 and HE4 to increase the diagnostic sensitivity up to 84% in stage I [36]. • Can be elevated in various cancers, including colorectal, [61], lung [62], gastric [63], endometrial [64] and breast cancer [65].
Kallikreins	Level more than 4.4 mg/L indicated poor prognosis in patients [66].	• Exhibit low sensitivity in the early detection of ovarian cancer. • It must be combined with CA-125 for higher specificity and sensitivity [67].
Osteopontin	Has a sensitivity of 83.3% in the detection of ovarian cancer [68].	• Its specificity is low. • It must be combined with CA-125 for higher sensitivity [69].
Mesothelin	Elevate in patients with ovarian cancer compared with normal healthy [70].	• Is not useful markers for early detection [71].
M-CSF	Elevated levels of M-CSF1 in serum and ascites are associated with a poor prognosis [72]. Serum M-CSF appears to improve the diagnostic reliability of serum CA 125 alone [73].	• This biomarker expressed also in other cancers [74].
Bikunin	Mediates suppression of tumor cell invasion and metastasis. Low expression is associated with late-stage disease. Low response to chemotherapy, and reduced survival time [44].	• Bikunin is present predominantly in amniotic fluid and urine of healthy individuals [75].
EphA2	Overexpression is associated with poor prognosis [45].	• EphA2 is overexpressed in many human cancers [76, 77].
Transthyretin	Efficient serum marker for the diagnosis [47].	• Plasma levels, affected by acute and chronic diseases. • Its usage must be considerate [78].
Transferrin receptor 1	Overexpression in high-grade tumor tissues [79].	• Overexpressed in several cancers [80–82].
B7-H4	Over expression can be used as a tumor marker with negative prognostic effect for epithelial cell ovarian cancer potential immunotherapeutic target [83].	• B7-H4 is highly expressed in various human tumors, including breast, ovarian, lung, pancreatic, gastric and urothelial cell carcinoma [84, 85].
Prostasin	Overexpress in ovarian cancer patients at levels significantly higher than normal controls [50].	• Many human cancers show unusual expression of prostasin like urinary bladder, uterine, prostate, gastric and ovarian cancers [86, 87].
EGF receptor	Is associated with less favorable disease outcomes [88].	• Little or no difference to survival, either as maintenance treatment after first-line chemotherapy or in association with chemotherapy in recurrent cancer [89].

*Abbreviations*: *CA125* cancer antigen 125, *HE4* human epididymis secretory protein 4, *VEGF* vascular endothelial growth factor, *M-CSF* macrophage colony stimulating factor, *EphA2* ephrin type-A receptor 2, *B7-H4* a molecule of B7 family

### Circulating tumor DNA (ctDNA)

**Fig. 1:**
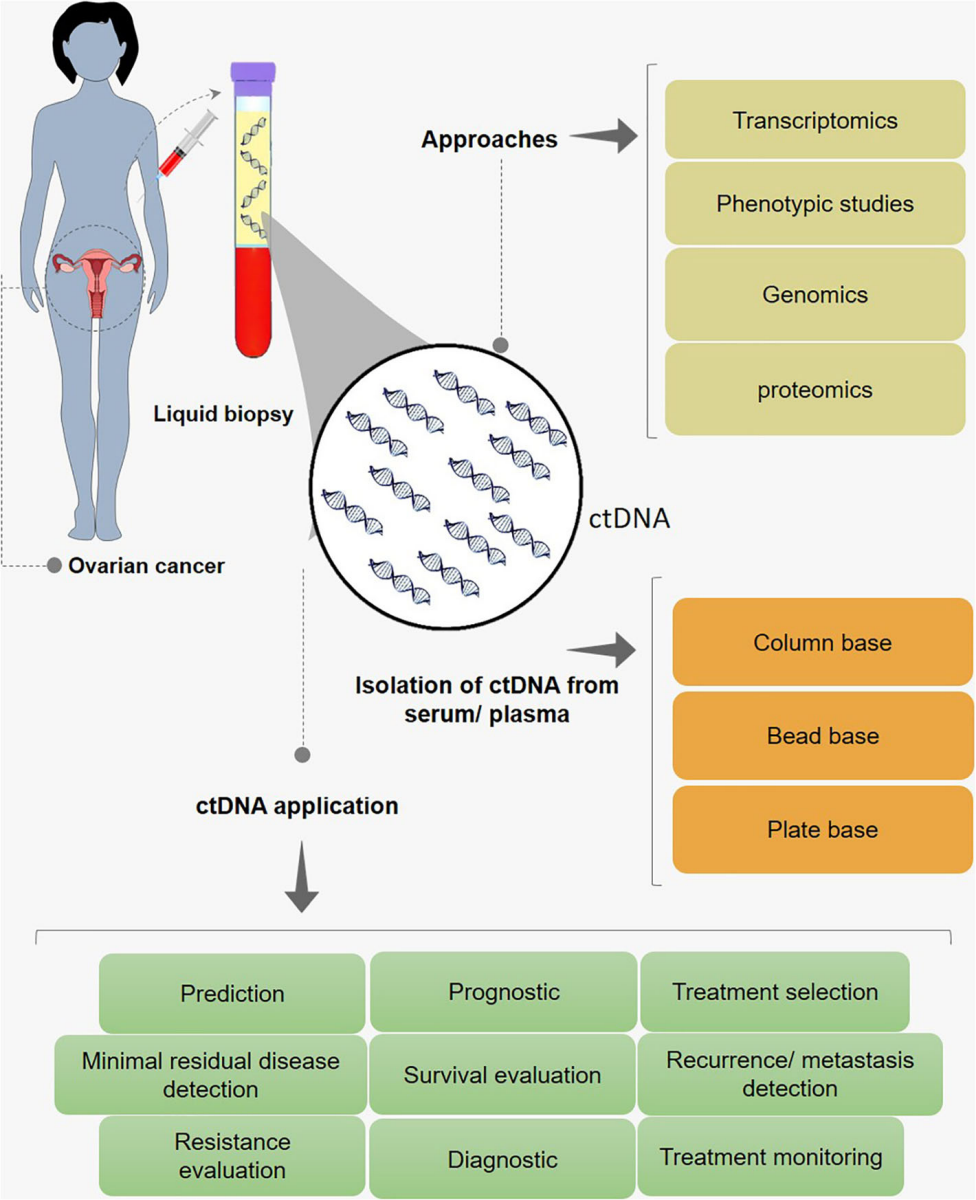
ctDNA isolation and application in the ovarian cancer patient

Liquid biopsy is a minimally invasive approach, which can be applied for the detection of molecular biomarkers from body fluids with no need for the costly or invasive procedures [90]. Accordingly, it is considered as a simple and non-invasive alternative to surgical biopsies, which enables discovering a wide range of information on a disease or a tumor through a simple blood sample. Notably, new dedicated methods allow us screening and monitoring cancer cell through circulating tumor cells (CTCs) and extracellular mirco-vesicles (including exosomes) containing small-RNA, mRNA, and ctDNA [91]. Tumor-associated genetic alterations can elucidate the molecular stratification of tumors toward the identification and selection of an appropriate targeted treatment. Historically, the presence of ctDNA in the blood of the patients with cancer was firstly recognized in the 1970s [92]. The elevated level of ctDNA is observed at the advanced stage of the disease progression, partly due to the reduced DNase activity [93]. Besides, ctDNA can reflect some specific genomic alterations of the tumor origin including mutations, methylation, and copy number variations (CNVs) [94] and preserving the genetic characteristics of the original tissue [95] (Fig. 1). The ctDNA-based mutation detection was also reported in 82% of the metastatic patients and 52% of the patients with localized disease [96]. Importantly, 95% concordance in mutational status was found between ctDNA and matched tumor tissue, which suggest that, the analysis of ctDNA, as a non-invasive approach, not only allows the tumor characterization and resolves the complexities of conventional tissue biopsy, but it also acts as an indicator for monitoring the treatment response in a given therapy [97]. A reliable biomarker can ensure that, which patients are more likely to relapse after receiving the adjuvant therapy [98]. Also, usage of ctDNA monitoring for a successful treatment over the resistance was reported in several clinical trials [99, 100]. Moreover, an invasive conventional tumor biopsy cannot be performed, while the frequent imaging can increase the risk of radiation exposure. Thus, the minimally invasive tests can be frequently repeated in a time series framework, which provide constant updates of tumor genetic composition and mutations, suggesting the best course of treatment at an appropriate time [101].

### Tumor tissue-based mutations versus ctDNA mutations in ovarian cancer

Over the last few years, genome-wide analysis revealed numerous alterations in ovarian cancer genomes including the inactivation of mutations in tumor suppressor genes such as *TP53, BRCA1, BRCA2*, *PTEN,* and *RB1*; and in a *SWI/SNF* chromatin remodeling gene, *ARID1A* [102, 103]. Other studies have detected the activation of mutations in the oncogenes *KRAS, PIK3CA, BRAF,* and *ERBB2* [102, 104–106]. In fact, identifying the common gene mutation in blood rather than tissue, can be helpful in determining the patients whom benefit from therapy using the existing molecular targeting drugs. In this regard, Morikawa et al., [107] using the droplet digital PCR (ddPCR) detected PIK3CA-H1047R and *KRAS*^*G12D*^ mutations in tumor tissue and also matched ctDNA of 33 patients with ovarian clear cell carcinoma and then monitored their response to therapy. Accordingly, they highlighted the detection of mutations in ctDNA as a powerful tool for the diagnosis of ovarian clear cell carcinoma and for predicting its recurrence. In addition to this, Ogasawara et al. [108] assessed *the PIK3CA* and *KRAS* mutations in tumor and ctDNA of 304 patients with ovarian cancer. In addition, they indicated that, the detection rates of *PIK3CA* and/or *KRAS* ctDNA mutations were associated with the advanced stage; however, they were not related to the histologic subtype or residual tumor status. ctDNA detection was also associated with the shorter progress free survival (PFS) and the increased risk of recurrence independently [108]. In a pre-clinical study, the somatic mutation status of the *TP53* was evaluated in both patient-derived tumor specimens and corresponding ctDNA, which resulted into the detection of similar hotspot mutation in *TP53* in both sources of biopsy [109]. Moreover, Yong-Man Kim et al. [110] assessed the *TP53* mutations across 103 tumor tissues from 61 patients with a high grade ovarian cancer and also confirmed *TP53* mutations in 41 patients. They concluded that, detection of *TP53* mutation in ctDNA is a potential tumor-specific biomarker for the treatment response monitoring [110]. Clinically, a copy number abnormality was reported in 20% of the patients with ovarian cancer [111] including claudin 4 (*CLDN4*), RAS oncogene family (*RAB25*), and ATP binding cassette subfamily F member 2 (*ABCF2*) [112, 113]. Hong No et al. [114] reported the lack of positive association between mutation in these genes at ctDNA level with the disease-free survival (DFS) and OS [114]. *Moreover, BRCA1* and *BRCA2* play pivotal roles in DNA repair and germline mutations [115]. Additionally, these *BRCA1/2* reversion mutations could be detected by ctDNA sequencing analysis in the patients who received platinum and/or PARP inhibitors [116]. Rebecca et al. [117] compared the genetic variants of a panel of 50 genes between tumor and ctDNA among 20 patients diagnosed with the high-grade ovarian carcinoma during neoadjuvant chemotherapy (NACT). Notably, 38 genetic variants out of six genes (*TP53, KIT, KDR, KRAS*, *PIK3CA,* and *PTEN*) were identified in tumors pre-NACT, while 59 variants out of 19 genes were detected in the ctDNA. In this study, targeted NGS determined the increased level of ctDNA variants with a minimal overlap between ctDNA and tumor DNA. Most of the mutations found in ctDNA were not present in tumor resulting from the amplifying of ctDNA. Besides, the heterogeneity in the tumor can be detected in ctDNA, in contrast with tumor tissue [117].

### The potential application of ctDNA to the management of treatment response in ovarian cancer

To date, FDA has not approved any ctDNA-related test in ovarian cancer. In this regard, a clinical ctDNA workflow was recently designed in the management of the high-grade ovarian cancer, to investigate the clinically actionable alterations of 500 cancer-related genes, which was performed in 12 patients. In seven patients, a good concordance of mutations and copy number alterations in ctDNA and tumor samples (*NF1, RAD51C, PTEN, BRCA2, STAG2, FANCA, CDKN1B, ERBB2, ERBB4, and MAP 2 K1*), and also alterations associated with the clinically available drugs (PI3K/mTOR inhibitor, PARP inhibitor, CDK2/4 inhibitor, CDK4/6 inhibitor, HER2 inhibitor, trastuzumab, ERBB inhibitor, lapatinib, and EGFR inhibitor) were detected. One chemo resistant patient therapy has changed based on the detection of *ERBB2* amplification and ctDNA-guided decision. These results can be considered as a proof of using ctDNA concept to guide the clinical decisions during the cycles of chemotherapy in ovarian cancers [118]. In addition, Noguchi et al. [119] compared the variant allele frequency (VAF) of the measured ctDNA mutations during neoadjuvant chemotherapy in 10 plasma samples. In 5 out of 6 NAC-sensitive cases, the VAF of non-synonymous somatic mutations (*TP53, KCAN5,* and *GJA8*) decreased following NAC. Also, in two out of the four NAC-resistant cases, the VAF increased in the non-synonymous somatic mutations (*KRAS, TRPS,* and *TP53*). The rate of TP53 mutation was significantly higher in the resistant cases compared with the sensitive cases. In addition, the blood tumor mutation burden significantly decreased after the treatment in the sensitive cases. These findings showed that, gene mutation can be profiled and then monitored using ctDNA in ovarian cancer patients during treatment. The Table 2 summarizes the studies that used ctDNA analysis to monitor treatment response in ovarian carcinoma. Lately, a multi-center prospective study demonstrated that, detectable ctDNA following treatment is associated with a subsequent recurrence in ovarian cancer (trial, NCT03691012). In this study, serial blood and tumor samples were collected from 100-stage I-IV debulked ovarian cancer patients under the platinum-based treatment. Each patient was followed for more than a 6- to 8-month period for ctDNA (mutation) and CA125 analysis. After the completion of 6 cycles of chemotherapy, analysis of plasma ctDNA has been shown to exhibit the tumor-related alteration. Regarding the treatment response monitoring in ovarian cancer, an ongoing prospective multicenter trial (NCT03302884) was established to assess ctDNA value for ovarian cancer recurrence after the front-line treatment of chemotherapy to profile the significant gene modifications before the clinical diagnosis of disease relapse. In addition, the assessment of the minimal residual disease through plasma ctDNA in the ovarian cancer patients is currently underway the prospective study (trial, NCT03614689). In this study, match tumor DNA and longitudinal plasma sample was collected from 100 ovarian cancer patients before, during, and after the treatment. The correlation between the clonal status of mutations and therapy response, whether ctDNA detection would be used to predict the ovarian cancer recurrence risk before and after treatment, is an important point that was considered in this study. The immune checkpoint blockade (ICB) recently provides clinical benefits to a subset of patients with ovarian cancer. Bratman et al. [120] have started a prospective phase II clinical trial to assess ctDNA in five distinct cohorts of patients including high grade serous ovarian cancer patients with advanced solid tumors treated with pembrolizumab (NCT02644369). At baseline, 316 serial plasma samples at every three cycles from 94 patients were obtained. These findings indicated that serial ctDNA analysis could serve as a general monitoring strategy for patients treated with ICB and correlates with their survival. The Table 3 summarizes the clinical trial studies that used ctDNA analysis in ovarian cancer management.

**Table 2:** Studies of ctDNA in ovarian cancer patients related to treatment response monitoring

References	year	No of patients	Identified Abnormalities	Methodology
Gifford et al. [121]	2004	138	hMLH1 methylation	Microsatellite PCR
Swisher et al. [122]	2005	137	p53 mutation	DNA sequencing
Kamat et al. [123]	2006	–	Level	RT-PCR
Capizzi et al. [124]	2008	22	Level	RT-PCR
Kamat et al. [125]	2010	164	Beta-globin	RT-PCR
Wimberger et al. [126]	2011	62	Fluorimetry	Fluorescence
Forshew et al. [127]	2012	38	*TP53. Other markers include PTEN, BRAF, KRAS, EGFR, PIK3CA*	TAm-Seq, dPCR
Murtaza et al. [128]	2013	3	*RB1, ZEB2, BUB1, CES4A, MTOR, PARP8*	NGS, qPCR
Choudhuri et al. [129]	2014	100	Level	RT-PCR
Martignetti et al. [130]	2014	1	*FGFR2* fusion transcript	RT-PCR
Pereira et al. [131]	2015	22	*TP53, PTEN, PIK3CA, MET, KRAS, FBXW7, BRAF*	WES, ddPCR, TGS
Cohen et al. [132]	2016	32	CNV	WES
Harris et al. [133]	2016	10	Aberrant chromosomal junctions	RT-PCR
Piskorz et al. [134]	2016	18	TP53 mutation	NGS
Parkinson et al. [135]	2016	40	TP53 mutation	Digital PCR
Vanderstichele [136]	2017	57	CNV	WGS
Phallen et al. [137]	2017	42	55 gene panel including *TP53, KIT, ALK, APC, ERBB4* etc	NGS (TEC-Seq) and ddPCR
Flanagan et al. [138]	2017	247	Methylation at CpG sites	NGS
Widschwendter et al. [139]	2017	151	Regions linked to *COL23A1, C2CD4D* and *WNT6*	TUC-BS & RRBS
Ratajska et al. [140]	2017	121	*BRCA1/2* mutations	NGS
Christie et al. [141]	2017	30	*BRCA* reversion mutation	NGS
Weigelt et al. [116]	2017	19	*BRCA* reversion mutation	NGS
Giannopoulou et al. [142]	2018	50	*ESR1*	RT-MSP
Du et al. [143]	2018	21	CNV and mutant genes including *TP53, BRCA1, NOTCH2, DNMT3A* etc	NGS
Morikawa et al. [107]	2018	29	*KRAS and PIK3CA*	ddPCR
Nakabayashi et al. [144]	2018	36	CNV	WGS
Park et al. [109]	2018	4	*TP53*	ddPCR
Arend et al. [117]	2018	14	50 gene panel	NGS
Lin et al. [145]	2019	97	BRCA reversion mutation, *TP53*	NGS
Kim et al. [110]	2019	102	TP53 mutant allele	Sanger sequencing/Digital PCR
Oikkonen et al. [118]	2019	12	*ERBB2* amplification	NGS
Iwahashi et al. [146]	2019	4	*TP53, APC, BRCA1 and KRAS*	CAPP-seq
Noguchi et al. [119]	2020	10	gene mutation profiles and blood tumor mutation burden	CAPP-seq
Han et al. [147]	2020	10	88 genes panel	NGS
Alves et al. [148]	2020	11	Level	qPCR

*Abbreviations*: *NGS* Next-generation sequencing, *RT-PCR* Reverse transcription polymerase chain reaction, *dPCR* droplet Polymerase chain reaction, *qPCR* Allele-specific quantitative PCR, *RT-MSP* Real-Time methylation specific PCR, *CNV* Copy number variation, *WGS* Whole genome sequencing, *WES* Whole exome sequencing, *ddPCR* Droplet digital PCR, *TGS* Targeted gene sequences, *TAm-RSeq* Targeted amplicon re-sequencing, *RRBS* Reduced representation bisulphite sequencing, *TUC-BS* Targeted ultra-high coverage bisulphite sequencing, *CAPP-seq* Cancer Personalized Profiling by deep Sequencing

**Table 3:** Clinical trial studies related to ctDNA in ovarian cancer patients

Clinical trial title	Participants	Date	Interventions	Recruitment Status	ClinicalTrials.gov Identifier
Plasma ctDNA detection in diagnosis of epithelial ovarian cancer. (ctDNA_EOC)	43	October 19, 2017	Diagnostic Test: methylation markers screening	Completed	NCT03155451
Study of circulating tumoral DNA in ovarian cancer.	25	January 23, 2017	Blood sampling	Completed	NCT01350908
Circulating tumor DNA guiding (Olaparib) Lynparza® treatment in ovarian cancer.	160	October 18, 2018	• Drug: Olaparib • Drug: carboplatin + gemcitabine or carboplatin + paclitaxel or carboplatin + liposomal doxorubicin or liposomal doxorubicin 4-weekly or topotecan or paclitaxel weekly	Recruiting	NCT02822157
Assessment of the minimal residual disease in ovarian cancer from circulating tumor DNA and immune repertoire.	100	August 3, 2018	–	Recruiting	NCT03614689
Circulating tumor DNA as a marker of residual disease & response to adjuvant chemotherapy in stage I-IV ovarian cancer.	100	October 1, 2018	Diagnostic Test: Circulating tumour DNA testing	Recruiting	NCT03691012
Circulating tumor DNA as an early marker of recurrence and treatment efficacy in ovarian carcinoma (CIDOC).	150	September 26, 2019	biological sampling	Recruiting	NCT03302884
Study of the effects of pembrolizumab in patients with advanced solid tumors	94	March 21, 2016	• Biological: Pembrolizumab	Active, not recruiting	NCT02644369

### Conclusion and future perspective

In summary, ctDNA detection before treatment facilitates early detection and leads to appropriate treatment decision-making based on patient stratification. Monitoring of the residual disease helps in prevention of recurrence of the tumor. During the course of treatment, regular monitoring of ctDNA can elucidate drug resistance acquired from genetic alterations, which are always present but not detectable by conventional approaches. Therefore, genomic-based drug response prediction can open new horizons in oncology to enable better cancer patient’s management. In addition, a considerable number of clinical trials, mentioned in Table 3, highlight the strong and novel roles of ctDNA in ovarian cancer management guidelines. Further efforts are required in the future for standardization of analysis platforms and incorporation of liquid biopsies as a companion biomarker in large-scale therapeutic trials.

## Data Availability

This manuscript is a review paper. So, “Not applicable”.
